# A Modified Arrhenius Approach to Thermodynamically Study Regioselectivity in Cytochrome P450‐Catalyzed Substrate Conversion

**DOI:** 10.1002/cbic.201900751

**Published:** 2020-02-25

**Authors:** Rosa A. Luirink, Marlies C. A. Verkade‐Vreeker, Jan N. M. Commandeur, Daan P. Geerke

**Affiliations:** ^1^ AIMMS Division of Molecular Toxicology Vrije Universiteit De Boelelaan 1108 1081 HZ Amsterdam The Netherlands

**Keywords:** cytochrome P450, molecular dynamics, selectivity, thermodynamics, validation

## Abstract

The regio‐ (and stereo‐)selectivity and specific activity of cytochrome P450s are determined by the accessibility of potential sites of metabolism (SOMs) of the bound substrate relative to the heme, and the activation barrier of the regioselective oxidation reaction(s). The accessibility of potential SOMs depends on the relative binding free energy (ΔΔ*G*_bind_) of the catalytically active substrate‐binding poses, and the probability of the substrate to adopt a transition‐state geometry. An established experimental method to measure activation energies of enzymatic reactions is the analysis of reaction rate constants at different temperatures and the construction of Arrhenius plots. This is a challenge for multistep P450‐catalyzed processes that involve redox partners. We introduce a modified Arrhenius approach to overcome the limitations in studying P450 selectivity, which can be applied in multiproduct enzyme catalysis. Our approach gives combined information on relative activation energies, ΔΔ*G*_bind_ values, and collision entropies, yielding direct insight into the basis of selectivity in substrate conversion.

## Graphical abstract

**Selectivity insight**: Measuring the activation energies of enzymatic reactions often involves analyzing reaction rate constants at various temperatures and constructing Arrhenius plots. This is a challenge for multistep cytochrome P450‐catalyzed processes that involve redox partners. Herein we present a modified Arrhenius approach to enable the study of thermodynamic determinants of selectivity in P450‐catalyzed substrate conversion.

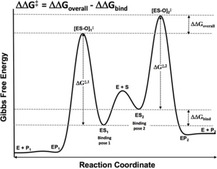

## Introduction

Cytochrome P450 enzymes (P450s) form a superfamily of heme‐containing proteins that play an important role in the oxidative metabolism of many lipophilic xenobiotics, as well as in the biosynthesis and catabolism of endogenous compounds.^1^ P450s are considered to be the catalytically most diverse enzymes in nature and because of their versatility they have many potential biotechnological applications.^2^, ^3^, ^4^, ^5^, ^6^, ^7^, ^8^, ^9^, ^10^, ^11^, ^12^, ^13^, ^14^, ^15^ So far, sequences of over 300 000 isoforms have been determined in all domains of life.^16^ In humans, P450s are responsible for approximately 75 % of phase I metabolism of currently marketed drugs and are involved in the activation of several prodrugs and toxicants.^17^ Therefore, there is great interest in predictive tools to determine the metabolic properties of P450s.

**Figure 1:**
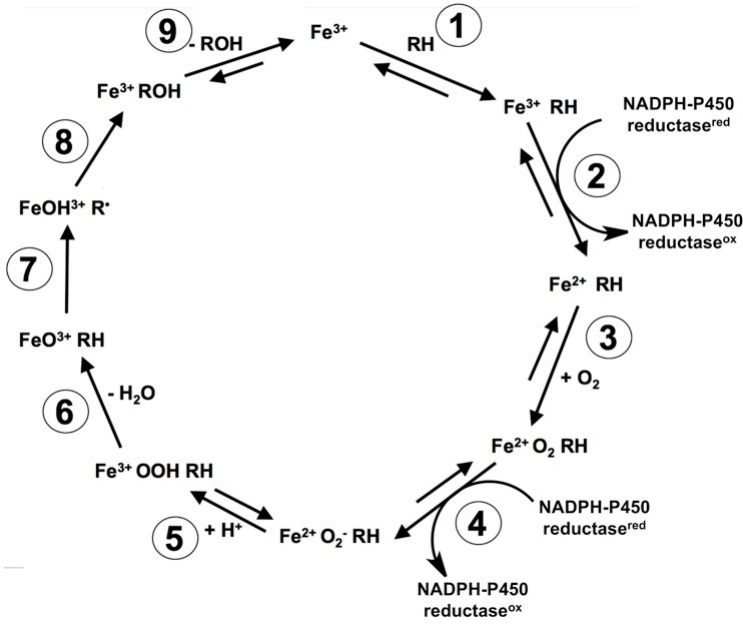
Catalytic cycle for cytochrome P450 catalyzed hydroxylation of substrate RH.

So far more than thirty different types of reactions are described for P450s.^18^−^20^ The predominantly occurring P450 reactions include C‐hydroxylation, heteroatom dealkylation, epoxidation and heteroatom oxidation. Figure 1 shows the catalytic cycle for P450‐mediated hydroxylation reactions consisting of 1) substrate binding, 2) one‐electron reduction of the ferric iron, 3) binding of molecular oxygen to the ferrous iron, 4) a second one‐electron reduction, 5) protonation of the Fe^2+^OO^−^, 6) heterolytic cleavage of the hydroperoxyl bond to yield FeO^3+^, 7) hydrogen abstraction of C−H‐bond, 8) rebound of hydroxy group, and 9) release of the product. Which step is rate limiting appears to depend on the specific combination of P450 isoform and substrate involved in the hydroxylation reaction.^21^, ^22^, ^23^, ^24^, ^25^, ^26^, ^27^, ^28^, ^29^, ^30^ For several substrates the rate‐limiting nature of hydrogen abstraction has been demonstrated by the kinetic isotope effect (KIE) observed after deuterium substitution.^23^, ^27^, ^30^

It is generally accepted that the regio‐ and stereoselectivity of P450s are governed by 1) the preference and probability of the substrate to bind in a reactive orientation relative to the activated oxygen species, and 2) the activation energies of the specific oxidation reactions at the exposed sites of metabolism (SOMs).^31^ To predict and/or rationalize regioselective metabolite formation, several in silico approaches have been used to study orientations in substrate binding and/or the activation energy (*E*_a_) of the oxidation reactions involved. Computational approaches to study the orientation(s) and dynamics of substrate binding include docking methods, molecular dynamics (MD) simulations, and/or binding free energy computation.^12^, ^13^, ^32^, ^33^, ^34^ Furthermore, quantum mechanical methods such as density functional theory (DFT),^35^, ^36^, ^37^, ^38^, ^39^, ^40^, ^41^, ^42^ albeit in combination with molecular mechanics (MM) techniques, have been used to calculate values for *E*_a_. There is still limited experimental data of preferred binding modes and activation energies to validate the predictivity of these computational approaches. Possible binding orientations of substrates in the active site of P450s have been studied experimentally using co‐crystallography^43^, ^44^, ^45^ and spin‐relaxation studies.^46^, ^47^, ^48^ However, in co‐crystallography studies the bound substrate sometimes appears too distant from the active center to be catalytically accessible.^44^, ^49^ In such cases, rearrangement of the substrate in the active site, which can be triggered by heme‐iron reduction, may well be required to adopt a productive complex.^50^, ^51^ In addition, resolving the electron density can be difficult when a substrate is able to bind in multiple orientations, which is not unusual for P450s.

A common experimental method to determine activation energies of chemical and enzymatic reactions is the quantification of the reaction rate constant for substrate‐to‐product conversion (*k*_cat_) at different temperatures, and by subsequently constructing logarithmic plots of (ln *k*_cat_) versus the reciprocal absolute temperature (1/*T*) according to the Arrhenius equation,^52^, ^53^

(1) $$k{}_{\mathrm{cat}}=Ae{}^{-E{}_{a}/RT}$$

According to Equation (1) the slope of the linear plot obtained equals the negative value of the activation energy *E*_a_ divided by the gas constant *R*. The pre‐exponential factor *A* comprises the frequency or collision efficiency at which the activated enzyme**⋅**substrate complex is formed. As such, it can be considered an entropic measure for the probability to form the transition state out of the enzyme**⋅**substrate complex.

**Scheme 1:**
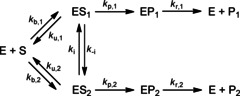
Kinetic scheme for the catalytic conversion of substrate S to two possible products, P_1_ and P_2_. The associated rate constants *k*_cat,1_ and *k*_cat,2_ depend on rate constants for the individual steps of the catalytic conversion, which comprise binding to (*k*_b_) and unbinding of (*k*_u_) the enzyme‐substrate complex ES, interconversion between ES_1_ and ES_2_ (*k*_i_, *k*_‐i_), formation of the enzyme‐product complex EP out of ES (*k*_p_), and unbinding of the EP complex (*k*_r_).

Until now, several examples of Arrhenius plots of P450 catalyzed reactions have been reported.^18^, ^54^, ^55^, ^56^, ^57^, ^58^, ^59^ These studies show that direct application of this or related approaches to P450s has several limitations. First, the slopes of the Arrhenius plots do not necessarily represent the activation energy of the oxidation reaction because steps prior to the oxidation reaction, such as reduction by the NADPH cytochrome P450 reductase (P450 reductase) and/or cytochrome b_5_ reductase (steps 2 and 4, Figure 1), may also be rate limiting for the overall reaction.^21^ Second, nonlinear Arrhenius plots were found with reactions catalyzed by microsomal P450s with a discontinuity at approximately 20 °C, which was attributed to a transition of membrane fluidity affecting the interaction between P450 and P450 reductase.^57^, ^58^ Furthermore, usually only a small range of temperatures is used because above the optimal temperature, the reaction rates decrease again due to enzyme denaturation. Finally, P450 catalysis often leads to different products at different ratios. Experimentally, *k*_cat_ values are usually determined by dividing values for the maximal velocity in substrate conversion as obtained from enzyme kinetic studies (*V*_max_) by the total enzyme concentration [E]_total_ (*k*_cat_=*V*_max_/[E]_total_), assuming that at maximal enzyme activity all enzymes are occupied by the substrate in a reactive binding pose. However, in the case of parallel reactions the individual *V*_max_ values cannot be divided by [E]_total_ but should be divided by the concentration of the enzyme‐substrate complexes of the corresponding reactive binding poses, designated [ES_1_] and [ES_2_] in Scheme 1 for possible formation of two products (P_1_ and P_2_). No direct experimental methods are available to accurately determine ratios of different bound conformations of a given enzyme**⋅**substrate complex.

In the present study we propose and evaluate a modified Arrhenius approach to overcome several of the above‐mentioned intrinsic limitations of thermodynamic studies on P450‐catalyzed reactions. In this modified approach the temperature dependence of the ratio of *V*_max_ values of parallel reactions is analyzed rather than studying the temperature dependence of kinetic parameters for individual pathways. Dividing the Arrhenius‐equations of the competing reactions in Scheme 1 (i.e., of reactions 1 and 2 with maximal velocities *V*_max,1_ and *V*_max,2_ and rate constants *k*_cat,1_ and *k*_cat,2_, respectively) and using *V*_max_=*k*_cat_×[ES]_max_ gives [Eq. (2)]:

(2) $$\frac{V{}_{\max,1}}{V{}_{\max,2}}=\frac{k{}_{\mathrm{cat},1}\times \left[\mathrm{ES}{}_{1}\right]{}_{\max}}{k{}_{\mathrm{cat},2}\times \left[\mathrm{ES}{}_{2}\right]{}_{\max}}=\frac{\left[\mathrm{ES}{}_{1}\right]{}_{\max}\times A{}_{1}}{\left[\mathrm{ES}{}_{2}\right]{}_{\max}\times A{}_{2}}\times e{}^{-(E{}_{a,1}-E{}_{a,2}/RT)}$$

The subscript max for concentrations of the enzyme‐substrate complexes indicates ES, ES_1_ or ES_2_ concentrations at maximal enzyme activity. Under a steady‐state approximation the ratio between [ES_1_] and [ES_2_] can be related to

(3) $$\frac{\left[\mathrm{ES}{}_{1}\right]{}_{\max}}{\left[\mathrm{ES}{}_{2}\right]{}_{\max}}=e{}^{-(\Delta \Delta G{}_{\mathrm{bind}}/RT)}$$

with ΔΔ*G*_bind_=Δ*G*_bind,1_−Δ*G*_bind,2_, which is the (possible) difference in binding free energies Δ*G*_bind_ between substrate binding poses associated with formation of products 1 and 2, respectively. The steady‐state approximation of Equation (3) is valid when substrate concentration [S]≫[E]_total_, and when the constants of substrate binding and unbinding (*k*_b_ and *k*_u_ in Scheme 1) are substantially higher than those for formation of the enzyme‐product (*k*_p,1_ and *k*_p,2_ in Scheme 1) and/or alternatively, when rapid interconversion between ES_1_ and ES_2_ is possible (with *k*_i_ and *k*_‐i_ in Scheme 1 being higher than the *k*_p_ values).

Hence, when correlating the natural logarithm of the ratio *V*_max,1_/*V*_max,2_ with the inverse absolute temperature (in a *modified* Arrhenius plot), a straight line is expected of which the slope of the plot will represent the sum (Δ) of the differences in activation energy (*E*_a_) and Δ*G*_bind_ of the parallel reactions (divided by the gas constant *R*), Equations (4), (5)

(4) $$\ln\left(\frac{V{}_{\max,1}}{V{}_{\max,2}}\right)=\ln\left(\frac{A{}_{1}}{A{}_{2}}\right)-\frac{\Delta \Delta G{}_{\mathrm{bind}}+\Delta E{}_{a}}{RT}$$

or

(5) $$\ln\left(\frac{V{}_{\max,1}}{V{}_{\max,2}}\right)=\ln\left(\frac{A{}_{1}}{A{}_{2}}\right)-\frac{\Delta }{RT}$$

where Δ=ΔΔ*G*_bind_+Δ*E*_a_, and Δ*E*_a_=*E*_a,1_−*E*_a,2._

Because we assume that the rates and temperature dependence of steps 2–6 of the catalytic cycle in Figure 1 will be similar for the parallel pathways, these factors will cancel out when evaluating the ratios of product formation and therefore will not contribute to differences in Δ in Equation  (5). In addition, changes in membrane fluidity (in case of microsomal P450s), suboptimal interaction between P450 and P450 reductases, and protein denaturation at increased temperatures are expected to affect *V*_max_ values of both pathways to a similar extent, which will allow studies over a larger temperature range.

**Figure 2:**
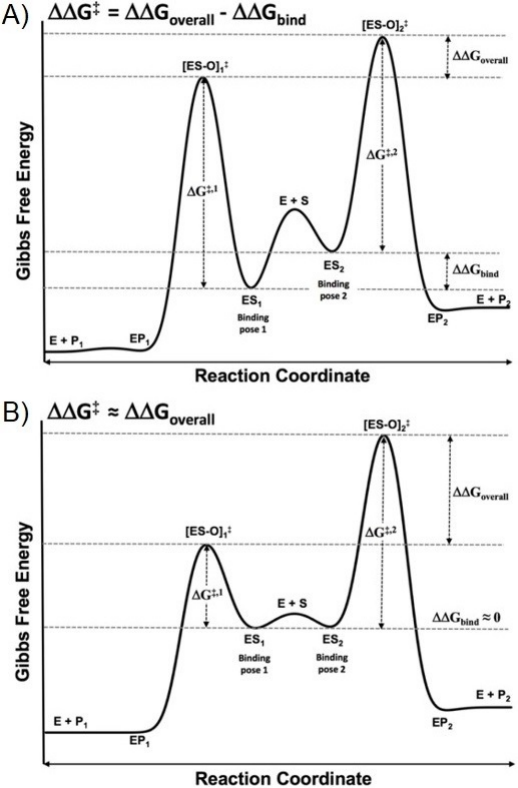
Gibbs free energy profile for a catalyzed reaction with two possible products A) with a binding free energy difference and B) without a binding free energy difference between the binding poses leading to the different products P_1_ and P_2_, starting from enzyme and substrate (E+S). Δ*G* ^≠,*x*^ comprises the sum of an activation energy (*E*_a,x_) and a collision entropy (*T*Δ*S* ^≠,*x*^) term.

In previous studies, ratios of product formation obtained at one incubation temperature have been used to estimate the overall difference in activation free energies (ΔΔ*G*_overall_) for competing reactions by P450s, Figure 2.^25^, ^60^ Also in this case, possible rate limiting factors prior to the oxidation reaction (steps 2–6, Figure 1) are expected to cancel out. According to the Curtin–Hammett principle,^61^ the product ratio of two competing reactions is governed by the difference ΔΔ*G*_overall_ between the free energies of the corresponding transition states ([ES‐O]_1_^≠^ and [ES‐O]_2_^≠^ in Figure 2) when the barrier to interconversion between reactive binding poses ES_1_ and ES_2_ (either direct or via enzyme/substrate unbinding) is much smaller than the barrier to product formation. The differences in the free energy of forming these transition states depend on the one hand on differences in free energies of binding of the productive binding poses ES_1_ and ES_2_ (ΔΔ*G*_bind_) and secondly by the differences between free energies of the binding poses and the transition states involved in product formation, with ΔΔ*G* ^≠^=Δ*G*_1_^≠^‐Δ*G*_2_^≠^, Figure 2 A [Eq (6)].

(6) $$\ln\left(\frac{\left[\mathrm{product}{}_{1}\right]}{\left[\mathrm{product}{}_{2}\right]}\right)=-\frac{\Delta \Delta G{}_{\mathrm{overall}}}{RT}=-\frac{\Delta \Delta G{}_{\mathrm{bind}}+\Delta \Delta G{}^{\neq }}{RT}$$

Previously, Higgins et al. assumed that when different human P450s show both similar kinetic isotope effects and product ratios, the product ratios observed are determined by the differences in activation free energy of the transition states ΔΔ*G* ^≠^ [Eq (7)]:^60^

(7) $$\ln\left(\frac{\left[\mathrm{product}{}_{1}\right]}{\left[\mathrm{product}{}_{2}\right]}\right)\approx -\frac{\Delta \Delta G{}^{\neq }}{RT}$$

This may well be valid for small substrates that can rapidly adopt multiple binding poses (high *k*_i_ and *k*_‐i_ in Scheme 1) and that have only a small difference in free energy of binding (ΔΔ*G*_bind_), *cf*. Figure 2 B. In such cases, ΔΔ*G* ^≠^ can be directly estimated from ratios in product formation using Equation (7). In the case of substrates with high molecular weight and/or P450s with restrictive active sites, next to differences in free energies of activation, steric factors may also play an important role in the regioselectivity of P450 reactions, and ΔΔ*G*_bind_ in Equation (6) cannot be neglected a priori. This is exemplified for example by recent binding free energy calculations^32^ for one of the pairs of product formation considered in the current work, and Equation (6) is in such cases to be used instead of Equation (7). The difference in using our modified Arrhenius approach compared to direct use of the Curtin–Hammett formalism is that the entropic contribution to differences in the barrier for forming the transition state from the enzyme‐substrate complex (i.e., the ratio A1/A2 in Equation (5)) can be separated from other contributions (ΔΔ*G*_bind_ and Δ*E*_a_). Therefore, our approach can be of direct help in validating (the combined use of) free energy, quantum chemical, and MD studies on preferred modes of substrate binding, activation energies, and/or probabilities to adopt catalytically active binding orientations, respectively.

**Figure 3:**
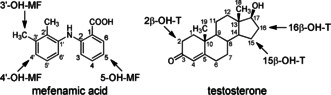
Structures of the compounds used in this study. Arrows indicate the sites of metabolism as experimentally observed for P450 BM3 mutant M11. Abbreviations refer to products formed by the regiospecific oxidation reactions. Note that mefenamic acid hydroxylation as catalyzed by P450 isoform 1A2 leads to formation of 4′‐OH‐MF and 5‐OH‐MF only.

In this study, the human P450 isoform 1A2 (CYP1A2) and a drug metabolizing mutant of bacterial P450 BM3 (CYP102A1), that is, BM3 M11, are used to evaluate the applicability and to illustrate the value of our approach to analyze thermodynamic determinants of selectivity in P450‐catalyzed product formation. For that purpose, we determined the temperature dependence of product ratios for pairs of different substrate conversions as catalyzed by the same isoform. Mefenamic acid (MF) and testosterone (TE) were selected as substrates. MF is oxidized by CYP1A2 and P450 BM3 M11 to two or three metabolites, respectively, while TE conversion catalyzed by BM3 M11 leads to three different products as well, Figure 3.^13^, ^62^ Recombinant CYP1A2 was selected as model for a membrane‐bound P450, which depends on co‐expressed NADPH cytochrome P450 oxidoreductase as redox partner. P450 BM3 M11 was used as model for a soluble P450. Wildtype P450 BM3 is a natural fusion protein between a P450 domain and P450 oxidoreductase domain and is often used for mechanistic studies of P450.^4^ Because it has the highest turnover recorded for any P450, it also has promising biotechnological perspective for biosynthesis of fine chemicals.^11^ Mutant P450 BM3 M11 was developed by combination of site‐directed and random mutagenesis and catalyzes oxidation reactions of a wide variety of pharmaceuticals and other chemicals.^6^ To determine Δ and relative collision efficiencies ln (*A*_1_/*A*_2_) in Equation (5) for the multiple substrate conversions catalyzed by CYP1A2 or P450 BM3 M11, enzyme kinetic parameters were determined for each reaction at different incubation temperatures. In support of our steady‐state approximation in Equation (3), we also measured kinetic isotope effects for the pair of product formation (i.e., conversion of MF to either 3′‐OH‐MF or 4′‐OH‐MF by BM3 M11, Figure 3) for which the corresponding ES binding poses were previously reported to be similar,^32^ and hence may well rapidly interconvert. In addition, molecular dynamics (MD) computer simulations of selected isoform‐substrate combinations were carried out to quantify the probability of the substrates to adopt different catalytically active binding poses, using geometric criteria for transition state formation based on combined QM/MM studies by Mulholland and co‐workers.^63^ The results were compared with the relative collision efficiencies as determined from the intercepts of our Arrhenius plots, and with differences between our estimated Δ values and corresponding Curtin–Hammett estimates for relative activation barriers, as measures for possible differences in the entropy of transition state formation. To further interpret and cross‐validate our modified Arrhenius and in silico analyses we also computed differences in activation energies *E*_a_ (using the SMARTCyp web server)^64^ and/or obtained them from literature, and where possible we combined these estimates with ΔΔ*G*_bind_ values reported in literature for a direct comparison with our values for Δ.

## Results and Discussion

### Temperature‐dependent mefenamic acid hydroxylation catalyzed by BM3 M11

**Figure 4:**
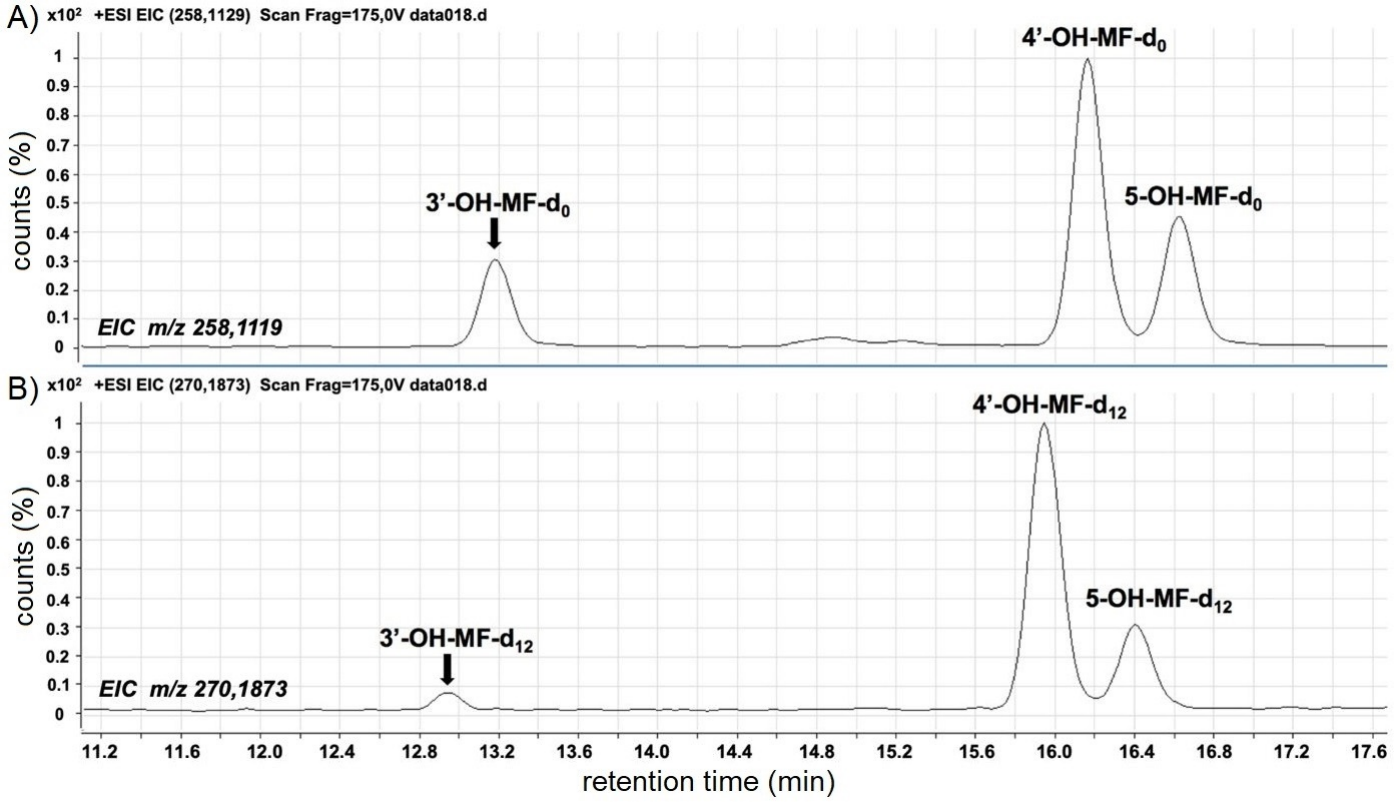
Extracted ion chromatograms of A) non‐deuterated (D_0_) and B) deuterated (D_12_) hydroxy metabolites of mefenamic acid, formed after incubations of P450 BM3 M11 with an equimolar mixture of deuterated (D_12/13_) and nondeuterated mefenamic acid (total concentration 750 μm), respectively.

As described previously,^33^ mefenamic acid was metabolized by P450 BM3 M11 to the three regioisomeric hydroxy metabolites shown in Figure 3. At all incubation temperatures 4′‐hydroxymefenamic acid (4′‐OH‐MF) was the major product, followed by 3′‐hydroxymethylmefenamic acid (3′‐OH‐MF), and 5‐hydroxymefenamic acid (5‐OH‐MF) as relatively minor product, Figure 4 A. As summarized in Table 1, the catalytic efficiency (*V*_max_/*K*_M_) for all three pathways increased from 4 to 25 °C. At higher temperatures the catalytic efficiency decreased again. Also for the *V*_max_ values the lowest values were obtained at the lowest and highest incubation temperatures. As a result, the plots of ln *V*_max_ versus 1000/*T* were strongly nonlinear when analyzing the kinetics of each metabolite individually (data not shown). This was expected based on previous Arrhenius studies on P450 catalyzed reactions.^56^, ^57^, ^58^, ^59^

**Table 1:** Temperature dependence of enzyme kinetic parameters for the regioselective hydroxylation of mefenamic acid by P450 BM3 M11 and recombinant human CYP1A2 (i.e., for the formation of the products 3′‐hydroxymethylmefenamic acid, 4′‐hydroxymefenamic acid and 5‐hydroxymefenamic acid).

3′‐Hydroxymethylmefenamic acid				4′‐Hydroxymefenamic acid			5‐Hydroxymefenamic acid		
*T* [K]	*K*_M_ [μm]	*V*_max_^[a]^	*V*_max/_ *K*_M_^[b]^	*K*_M_ [μm]	*V*_max_^[a]^	*V*_max/_ *K*_M_^[b]^	*K*_M_ [μm]	*V*_max_^[a]^	*V*_max/_ *K*_M_^[b]^
***P450 BM3 M11***									
277.0	72±16	135±10	1875	76±17	230±19	3026	77±22	24±3	312
283.4	140±18	212±9	1514	151±15	343±12	2271	124±19	34±2	274
290.2	161±14	561±44	3484	170±17	859±82	5053	148±15	85±8	574
292.7	145±45	498±33	3434	159±52	736±52	4628	188±37	72±4	383
298.6	85±20	463±33	5447	88±23	608±50	6909	86±25	64±7	744
304.4	266±56	607±53	2281	354±34	787±41	2223	409±165	81±7	198
313.3	556±200	358±28	644	603±150	431±37	714	384±202	42±5	109
317.6	388±184	194±19	500	538±34	208±14	387	762±118	21±2	27.6
***CYP1A2***									
279.4	N.A.	N.A.	N.A.	204±118	0.56±0.13	2.75	6±2	0.50±0.02	83.3
287.7	N.A.	N.A.	N.A.	210±92	0.99±0.17	4.71	47±21	0.78±0.09	16.6
299.4	N.A.	N.A.	N.A.	144±68	0.91±0.18	6.88	42±7	0.67±0.03	16.0
309.8	N.A.	N.A.	N.A.	182±33	1.0±0.09	5.49	47±4	0.68±0.02	14.5

[a] Unit: (nmol product) min^−1^ (nmol enzyme)^−1^. [b] Unit: μL min^−1^ (nmol enzyme)^−1^. N.A.: not applicable.

Before applying our modified Arrhenius analysis to the (three) pairs of BM3 M11 catalyzed product formations (Table 2), we measured relative kinetic isotope effects in an attempt to explicitly verify the steady‐state approximation taken in Equation (3) for the ratio of MF conversion to 3′‐OH‐MF and 4′‐OH‐MF. Representative extracted ion chromatograms of the mixed deuterated and non‐deuterated hydroxy metabolites in Figure 4 B show that the relative peak area of the 3′‐OH‐MF product (as compared with the peak areas for 4′‐OH‐MF or 5‐OH‐MF) decreases significantly when going from non‐deuterated to deuterated mefenamic acid. Table 3 summarizes that deuteration of mefenamic acid results in an approximately fourfold decrease in the rate of 3′‐methyl hydroxylation but to a 50 % increase of the 4′‐hydroxylation pathway, and no change in 5‐OH mefenamic acid formation. These results indicate that the hydrogen abstraction of the 3′‐methyl group is rate limiting to a more significant extent than aromatic hydroxylation reactions. This is in line with previous studies in which substantially larger KIEs were observed for aliphatic than for aromatic hydroxylation by P450s.^30^ The increase in 4′‐OH‐MF formation after deuteration may be well explained by metabolic switching resulting from the strongly decreased 3′‐hydroxylation. Thus, our kinetic isotope effect measurements for these reactions suggest that rapid interconversion between catalytically active poses for 3′‐OH‐MF and 4′‐OH‐MF formation is possible and accordingly, that *k*_i_ and *k*_−i_ values for binding‐pose interchange and/or *k*_b_ and *k*_u_ values are probably higher than *k*_p_ for the corresponding product formations. Therefore, even in the unexpected case that *k*_b_≪*k*_p_, the steady‐state approximation used in Equations (3)–(5) can still be assumed to be valid.

**Table 2:** Comparison of thermodynamic properties for the oxidation reactions of mefenamic acid and testosterone catalyzed by cytochrome P450 BM3 M11 and recombinant human CYP1A2, as determined using our modified Arrhenius approach [Eq. (5)], the Curtin–Hammett principle [Eq. (6)], or SMARTCyp (to calculate differences in activation energies, Δ*E*_a_).

Comparison for	Enzyme	ln (*V*_max,1_/*V*_max,2_) vs. 1000/*T*			Modified	Curtin–Hammett	SMARTCyp
the formation of:		Intercept	Slope [K]	*R* ^2^	Arrhenius: Δ	ΔΔ*G*_overall_	Δ*E*_a_
					[kJ mol^−1^]	[kJ mol^−1^]	[kJ mol^−1^]^[a]^
**Mefenamic acid**							
3′‐OH‐MF vs. 4′‐OH‐MF	BM3 M11	3.0±0.2	−0.97±0.05	0.97	8.1±0.5	0.7	−1.8
3′‐OH‐MF vs. 5‐OH‐MF	BM3 M11	5.3±0.2	−0.97±0.07	0.98	8.1±0.6	−4.9	−1.8
4′‐OH‐MF vs. 5‐OH‐MF	BM3 M11	2.3±0.2	0.00±0.07	0.14	0.0±0.6	−5.6	0
4′‐OH‐MF vs. 5‐OH‐MF	CYP1A2	2.8±0.3	−0.74±0.10	0.97	6.1±0.8	−0.8	0
**Testosterone**							
15β‐OH‐T vs. 16β‐OH‐T	BM3 M11	7.6±0.5	−1.95±0.14	0.99	16.7±1.1	−2.8	0
2β‐OH‐T vs. 16β‐OH‐T	BM3 M11	9.1±0.5	−2.82±0.16	0.99	23.5±1.3	0.8	−9.5
2β‐OH‐T vs. 15β‐OH‐T	BM3 M11	1.4±1.0	−0.87±0.29	0.82	7.2±2.5	3.6	−9.5

[a] SMARTCyp activation energies: 3′‐OH‐MF, 66.4 kJ mol^−1^; 4′‐OH‐MF, 68.2 kJ mol^−1^; 5‐OH‐MF, 68.2 kJ mol^−1^; 2β ‐OH‐T, 66.4 kJ mol^−1^; 15β ‐OH‐T, 75.9 kJ mol^−1^; 16β ‐OH‐T, 75.9 kJ mol^−1^.

**Table 3:** Kinetic isotope effects for hydroxylation of mefenamic acid by P450 BM3 M11: ratios of non‐deuterated (D_0_) vs. deuterated (D_11/12_) hydroxy metabolites formed in incubations of P450 BM3 M11 with an equimolar mixture of deuterated (D_12/13_) and non‐deuterated mefenamic acid (total concentration 750 μm).

*T* [K]	3′‐OH‐MF	4′‐OH‐MF	5‐OH‐MF
278	3.84±0.20	0.65±0.002	1.08±0.02
288	4.35±0.20	0.66±0.001	1.02±0.03
298	3.78±0.48	0.65±0.002	1.06±0.01
308	3.58±0.55	0.79±0.006	1.00±0.01

**Figure 5:**
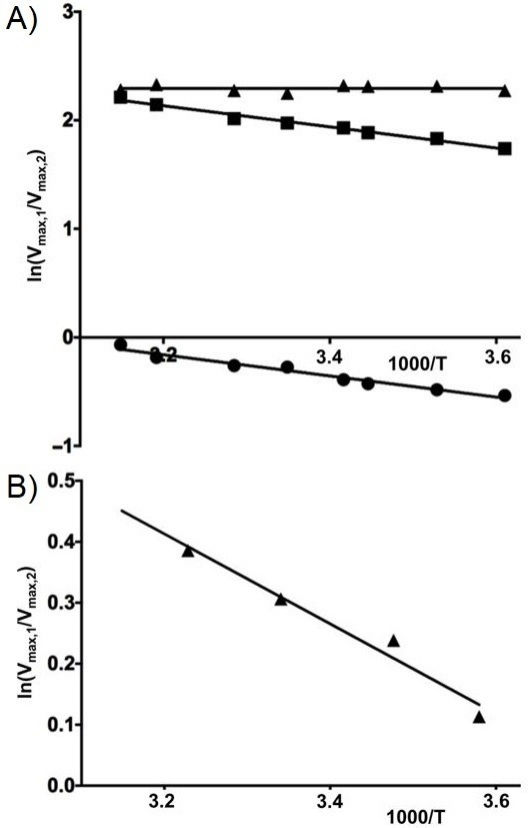
Modified Arrhenius plots of product ratios of mefenamic acid formed by A) P450 BM3 M11 and B) recombinant human CYP1A2, for the ratio ln (*V*_max,4′OH‐MF_/*V*_max,5‐OH‐MF_) (▴), ln (*V*_max,3′OH‐MF_/V_max,5‐OH‐MF_) (▪), and ln (*V*_max,3′OH‐MF_/*V*_max,4′‐OH‐MF_) (•).

In the next step we used Equation (5) to study the temperature dependence of the ratios in MF conversion by BM3 M11. When plotting natural logarithms of the ratios of *V*_max_ values of mefenamic acid metabolites against 1000/*T*, linear curves were obtained, Figure 5 A. This observed correlation supports our assumption that the temperature dependence of steps 2–6 of the catalytic cycle in Figure 1 will be similar for the different hydroxylation paths and cancel when relating *V*_max_ ratios to the inverse temperature. The slopes (Δ=ΔΔ*G*_bind_+Δ*E*_a_) and intercepts of the thus obtained modified Arrhenius plots are summarized in Table 2 and discussed below in our thermodynamic analysis of the observed regio‐specificity in MF conversion. In addition, ΔΔ*G*_overall_ values as derived (using the Curtin–Hammett principle and Equation (6)) from pairs of *V*_max_ values at 300 K are also reported in Table 2, as well as differences in activation energies Δ*E*_a_ calculated by using SMARTCyp. Note that we used two versions of SMARTCyp (versions 2 and 3), which gave identical results for *E*_a_. The small differences in activation barrier for all three hydroxylation reactions are in line with the similar values for *E*_a_ calculated at the B3LYP level of Density Functional Theory (DFT) by Leth et al.^65^ Leth modeled compound I as a porphyrin moiety without side chains and with axial coordinating O_2_^−^ and CH_3_S^−^ ligands, and showed for example a difference in the range of −6 to 3 kJ mol^−1^ in activation barrier when comparing 3′‐methyl‐OH‐MF and 4′‐OH‐MF formation. This is to be compared with the corresponding SMARTCyp value of −2 kJ mol^−1^ (Table 2).

For the ratio between 3′‐methyl‐OH‐MF and 4′‐OH‐MF formation the modified Arrhenius plot (Figure 5 A) shows a slope of −0.97±0.05 K which corresponds to a value for Δ [in Eq. (5)] of 8.1±0.5 kJ mol^−1^, with a lower sum of Δ*G*_bind_ and *E*_a_ for hydroxylation at the 4′ aromatic SOM, Table 2. As stated previously, Δ*E*_a_ is close to 0 (and even slightly negative); therefore the positive Δ value should be interpreted to result from a more negative (favorable) binding free energy for the catalytic binding orientation for 4′ hydroxylation of mefenamic acid as compared to 3′‐methyl hydroxylation (ΔΔ*G*_bind_=Δ−Δ*E*_a_=8.1−1.8=9.9 kJ mol^−1^), in agreement with and confirming the corresponding ΔΔ*G*_bind_ values previously computed by us that range between 9.3 and 11.6 kJ mol^−1^.^32^

Despite the lower binding affinity of MF to BM3 M11 in its pose that is catalytically active for 4′‐OH‐MF formation, the difference with 3′‐methyl hydroxylation in the overall activation free energy ΔΔ*G*_overall_ is close to zero from our Curtin–Hammett analysis (0.7 kJ mol^−1^, Table 2). Thus, the difference in Δ*G*_bind_ is for a large part counterbalanced by a lower entropy penalty to form the transition state for 3′‐methyl‐OH‐MF formation out of the corresponding ES complex, as reflected by the higher collision efficiency for 3′‐methylhydroxylation (with a value of 3.0±0.2 for the intercept of the modified Arrhenius plot, Table 2). We could cross‐validate these findings with MD simulations of BM3 M11 in complex with MF, in which we compared the frequencies of occurrence of MF binding poses that can potentially adopt transition state geometries for either 3′‐Me or 4′ hydroxylation. Indeed, our simulations showed higher frequencies for substrate orientations that are in line with the transition state geometries for 3′‐Me‐OH‐MF than for 4′‐OH‐MF product formation, Table 4. The fact that 4′ hydroxylation of MF by BM3 M11 is overall favorable over 3′‐methyl hydroxylation, despite the higher entropic cost for transition state formation and the slightly higher activation energy, should thus be understood in terms of the lower binding affinity for substrate binding in a pose that leads to 4′‐OH‐MF formation. Our previous detailed free energy perturbation computation study^32^ on selectivity in MF hydroxylation came to this conclusion as well and could thus be verified with our modified Arrhenius approach.

**Table 4:** Percentages of substrate binding orientations during two independent MD simulations of mefenamic acid bound to BM3 M11 (A and B) that are suitable for transition state formation for hydroxylation of mefenamic acid at its 3′‐methyl, 4′‐ or 5‐position.

	3′‐methyl	4′	5
A	79.2	66.1	29.0
B	76.6	46.7	16.0

For the ratio between 3′‐methyl‐OH‐MF and 5‐OH‐MF formation the modified Arrhenius plot (Figure 5 A) shows a slope of −0.97±0.07 K which corresponds to a value for Δ (in Equation (5)) of 8.1±0.6 kJ mol^−1^, with a lower sum of Δ*G*_bind_ and *E*_a_ for hydroxylation at the 5 aromatic SOM, Table 2. When considering the similar activation energy for 5‐OH‐MF as predicted by SMARTCyp (with Δ*E*_a_=−1.8 kJ mol^−1^, Table 2), the binding free energy should be higher for the 3′‐methyl binding pose, suggesting that binding in an orientation that can lead to 5‐OH‐MF formation is more favorable. As an alternative explanation, the difference in Δ might be due to a lower *E*_a_ value for 5′‐hydroxylation, as indicated by additional DFT calculations of Leth et al. in which dispersion corrections were explicitly included (i.e. using the B3LYP‐D3 level of theory), resulting in lower *E*_a_ value for 5‐OH‐MF formation by 14 kJ mol^−1^.^65^ In any case, from Table 2 the preference of 3′‐methyl over 5‐OH mefenamic acid formation by P450 BM3 M11 can be understood in terms of the higher probability (lower entropic cost) of transition state formation for 3′‐methyl hydroxylation, as indicated by the higher intercept of our modified Arrhenius plots and the observed difference between Δ and the Curtin–Hammett estimate for ΔΔ*G*_overall_. This observed difference is equal to *T*ΔΔ*S* ^≠^ under the assumption that trends in enthalpy and energy are equal, cf. Equation (8) and Figure 2.

(8) $$\Delta -\Delta \Delta G{}_{\mathrm{overall}}\;=\left(\Delta \Delta G{}_{\mathrm{bnid}}+\Delta E{}_{a}\right)-\left(\Delta \Delta G{}_{\mathrm{bind}}+\Delta \Delta G{}^{\neq }\right)=\Delta E{}_{a}-\left(\Delta \Delta H{}^{\neq }-T\Delta \Delta S{}^{\neq }\right)≅T\Delta \Delta S{}^{\neq }$$

This is in accord with our in silico data from refs. 32 and 33 showing strong hydrogen bonding interaction between mefenamic acid's carboxylate group and BM3 M11′s Ser72 residue, which directs substrate‐binding orientations for 3′‐methyl hydroxylation to adopt a catalytically active pose. Such an anchoring hydrogen bond is not present when bound in a pose enabling hydroxylation at the 5 position. Also in our MD simulations this particular hydrogen bond was observed in the simulations with mefenamic acid in the 3′‐methyl hydroxylation pose (Figure S1), which was not observed in the 5‐hydroxylation pose (Figure S2). Furthermore, we indeed observe a substantially higher frequency in MD simulations of substrate orientations corresponding to transition state formation for the 3′‐methyl hydroxylation compared to 5 hydroxylation (Table 4).

For the ratio between 4′‐OH‐MF and 5‐OH‐MF formation by BM3 M11, the modified Arrhenius plot (Figure 5 A) shows a slope of 0.00±0.07 K which corresponds to a value for Δ [in Eq. (5)] of 0.0±0.6 kJ mol^−1^, Table 2. The predicted identical *E*_a_ values for both pathways (Table 2) thus suggest a similar binding free energy for the corresponding catalytically active poses (ΔΔ*G*_bind_=Δ−Δ*Ε*_a_) whereas the lower B3LYP‐D3 value of Leth et al. (by 11 kJ mol^−1^)^65^ hints at preferred binding in the pose enabling 4′ hydroxylation. The preference of 4′ over 5 hydroxylation can again be understood in terms of the higher probability (lower entropic cost) of transition state formation for 4′ hydroxylation compared to 5‐OH product formation (Table 2), probably also due to hydrogen bonding with Serine 72 in the catalytic‐active binding pose in the former case (Figure S3).

### Temperature‐dependent mefenamic acid hydroxylation catalyzed by CYP1A2

Oxidation of mefenamic acid by recombinant CYP1A2 resulted in formation of 4′‐OH‐MF and 5‐OH‐MF. At lower substrate concentrations 5‐OH‐MF was the major metabolite, as indicated by the higher *V*_max_/*K*_M_ values, Table 1. *V*_max_ values of the 4′‐hydroxylation pathway were slightly higher than for 5‐hydroxylation. Nonlinear Arrhenius plots are obtained when plotting ln *V*_max_ versus 1000/*T* for the metabolites individually (data not shown). As for the pairs of BM3 M11 mediated product formations, the modified Arrhenius plot of the ratio of *V*_max_ values for the CYP1A2 catalyzed pathways as plotted against 1000/*T* showed linear behavior, Figure 5 B.

A higher collision frequency for formation of the transition state for 4′ hydroxylation (compared to 5‐hydroxylation, cf. the positive intercept and positive difference between Δ and ΔΔ*G*_overall_ in Table 2) suggests an entropically more favorable transition state formation for 4′‐OH‐MF formation. This is in line with the higher frequency observed in MD of substrate orientations corresponding to transition state formation for 4′ hydroxylation (Table 5). During the simulations, we observe a hydrogen bond between mefenamic acid and Thr469 when binding in the 5‐hydroxylation position (Figure S4), whereas we do not observe any stabilizing or positioning hydrogen bonds in the 4′‐hydroxylation binding pose (Figure S5). The slight preference of 5‐OH mefenamic acid formation by CYP1A2 (as reflected by the slightly negative ΔΔ*G*_overall_ estimate in Table 2) can in this case be associated to a lower binding free energy for and preferred binding in the corresponding catalytically active pose. Furthermore, the *E*_a_ value for 5′‐hydroxylation may be lower (see above).

**Table 5:** Percentages of substrate binding orientations during two independent MD simulations of mefenamic acid bound to CYP1A2 (A and B) that are suitable for transition state formation for hydroxylation of mefenamic acid at its 4′‐ or 5‐position.

	4′	5
A	33.1	1.9
B	65.1	7.2

### Temperature‐dependent testosterone hydroxylation catalyzed by P450 BM3 M11

**Figure 6:**
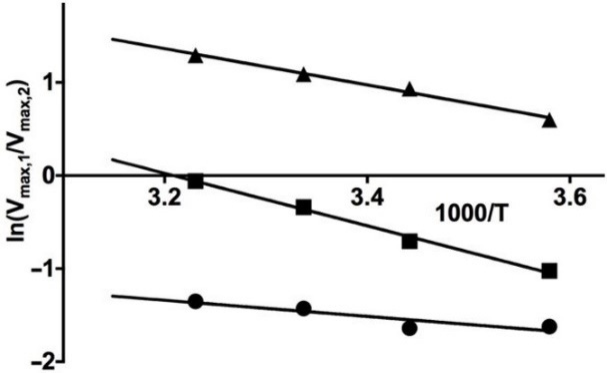
Modified Arrhenius plots of product ratios for testosterone conversion by BM3 M11, for the ratios ln (*V*_max,15β‐OH‐T_/*V*_max, 16β‐OH‐T_) (▴), ln (*V*_max,2β‐OH‐T_/*V*_max, 16β‐OH‐T_) (▪), and ln (*V*_max, 2β‐OH‐T_/*V*_max, 15β‐OH‐T_) (•).

As illustrated in Figure 3, testosterone can be hydroxylated by P450 BM3 M11 at three positions, leading in order of their relative amounts to 15β‐OH‐T, 16β‐OH‐T or 2β‐OH‐T formation, respectively.^46^, ^62^ The enzyme kinetic parameters of the reactions performed at temperatures ranging from 6 to 36 °C are shown in Supporting Information Table S1. As was observed for mefenamic acid, no linear Arrhenius plots are obtained when plotting ln *V*_max_ versus 1000/*T* for the metabolites individually (data not shown). Encouragingly, the modified Arrhenius plots of the ratios of *V*_max_ values versus 1000/*T* showed again linear behavior for all three combinations of pathways, Figure 6.

Using the slopes of these curves, Δ values for the three different pairs of product formation were obtained, Table 2. The minor pathway leading to 2β‐OH‐T was found to have the highest sum of Δ*G*_bind_ and *E*_a,_ with Δ=23.5 kJ mol^−1^ for the ratio with 16β‐OH‐T, and 7.2 kJ mol^−1^ for the ratio with 15β‐OH‐T. Thus, Δ is significantly higher for 2β‐OH‐T when compared with the other products, whereas the activation energy for formation of the corresponding transition state is lowest. This is apparent from the value for *E*_a_, which was predicted by SMARTCyp to be 9.5 kJ mol^−1^ lower than for 15β and 16β hydroxylation (Table 2), probably due to the adjacent C=O moiety beside of the C_2_ carbon. From previous comparative DFT calculations on the energy of C−H bond breaking we even found differences of more than 20 kJ mol^−1^ in favor of C−H bond activation at position 2 compared with positions 15 and 16.^66^ The higher Δ and lower *E*_a_ values for 2β‐OH‐T imply a significantly higher binding free energy (i.e., lower binding affinity) for TE binding in a pose compatible with 2β hydroxylation than for the other pathways, which may explain why it was difficult to find suitable starting poses from docking to start MD from of BM3 M11 with TE bound in this binding pose.

The Δ value for the ratio of formation of the major metabolite 15β‐OH‐T and the less abundant 16β‐OH‐T is 16.7 kJ mol^−1^, Table 2. *E*_a_ is probably higher for 15β hydroxylation than for 16β hydroxylation due to the substrate's hydroxy group at C17, which was also indicated by our previous finding that the energy cost of breaking the C15‐H and C16‐H aliphatic bonds is 5 kJ mol^−1^ higher for the former.^66^ In this particular case, the difference in *E*_a_ might be the only contribution to Δ as our MD simulations suggest that interconversion between binding orientations suited for 15β‐ and 16β‐hydroxylation can occur on the ns time scale. This is illustrated in Table 6 which shows that the geometric criteria for adopting the transition states for the 15β and 16β hydroxylation pathways can be both fulfilled during a single simulation. Therefore, information on the frequency of MD configurations consistent with transition state formation was for both positions taken from the same set of (three) simulations. The fact that 15β‐OH‐T formation is prevalent over 16β hydroxylation can be explained in terms of a higher collision frequency and entropy of transition state formation out of the enzyme‐substrate complex, as can be observed from the relatively large intercept in the modified Arrhenius plot for the ratio of 15β/16β hydroxylation and from the according difference between Δ and ΔΔ*G*_overall_, Table 2. These results are in line with the higher frequency observed in MD of substrate orientations corresponding to transition state formation for 15β hydroxylation (Table 6).

**Table 6:** Percentages of substrate binding orientations during three independent MD simulations of testosterone bound to BM3 M11 (A, B and C) that are suitable for transition state formation for hydroxylation of testosterone at its 15β or 16β position.

	15β	16β
A	49.7	4.5
B	49.7	16.2
C	40.3	53.1

## Conclusions

We have presented a method that makes it possible to obtain experimental estimates for thermodynamic determinants of regio‐ (and/or stereo‐)selectivity in P450 catalyzed substrate conversion, by means of studying temperature dependent ratios of pairs of metabolite formation as catalyzed by a single P450 isoform. We illustrated the use of this modified Arrhenius approach by studying the determinants of the regioselectivity in mefenamic acid and testosterone hydroxylation by P450 BM3 M11 and CYP1A2. For the selected P450‐substrate combinations, our approach gave insight into the basis of selectivity by giving combined information on relative activation energies, ΔΔ*G*_bind_ values, and collision entropy differences. We cross validated the observed collision entropy differences with molecular dynamics simulations, and we were able to verify previous computational free energy calculations. The obtained agreement suggests that the presented method can also be applied to other combinations of P450 isoforms and substrates that involve formation of two or more products. The methods and experiments described here are useful tools for future research on regio‐ and stereoselectivity of P450 catalysis to ultimately improve biocatalysts, and the data that can be obtained with our method allow to validate results from computational models to understand and predict selectivity.

## Experimental Section

**Materials**: Mefenamic acid, testosterone, NADPH, glucose 6‐phosphate and glucose‐6‐phosphate dehydrogenase were purchased from Sigma–Aldrich (Steinheim, Germany). Supersomes containing recombinant human P450s were obtained from BD Biosciences (Breda, Netherlands). The plasmid containing P450 BM3 M11 was constructed as described earlier.^6^ All other chemicals were of analytical grade and obtained from standard suppliers.

**Expression of cytochromes P450 BM3 M11 and P450 1A2**: His‐tagged cytochrome P450 BM3 M11 was expressed by transforming competent *Escherichia coli* BL21 cells with the corresponding pET28a+ vector and purified using nickel affinity chromatography as described previously.^62^ The method of Omura and Sato was used to determine the cytochrome P450 concentration.^67^

A bicistronic plasmid containing the cDNA of human CYP1A2 cDNA and human NADPH cytochrome P450 reductase was transformed into *E. coli* strain DH5α. A 300 mL terrific broth (TB) supplemented with 1 mm δ‐aminolevulinic acid, 0.5 mm thiamine, 400 μL L^−1^ trace elements, 100 μg mL^−1^ ampicillin, 1 mm isopropyl‐β‐d‐thiogalactopyranoside (IPTG), 0.5 mm FeCl_3_ was inoculated with a 7.5 mL pre‐culture grown from a single colony. The cells were allowed to grow for 40 h at 28 °C and 125 rpm. *E. coli* cells were collected by centrifugation (4000 *g*, 4 °C, 15 min) and resuspended in 20 mL 0.1 m potassium phosphate buffer at pH 7.4 containing 20 % glycerol, *v*/*v*, 0.25 mm ethylenediaminetetraacetic acid (EDTA) and 0.1 mm dithiothreitol (DTT). The cells were treated with 0.5 mg mL^−1^ lysozyme for one hour at 4 °C and subsequently disrupted by three cycles of disruption by Emulsiflex C3 emulsifier. The membranes containing the CYP1A2 were isolated by ultracentrifugation for 75 min at 40 000 rpm (169 936 *g*) and 4 °C. The pellet was resuspended in the potassium phosphate‐glycerol buffer and subsequently homogenized by Potter‐Elvehjem. The concentration of CYP1A2 was determined using the method of Omura and Sato^67^ and the enzyme was stored at −80 °C until use.

**Assessment of enzyme kinetic parameters at different temperatures**: All incubations were performed at enzyme concentration and incubation times for which the product formation was linear in time and proportioned to enzyme concentration (data not shown). The incubation mixtures contained 50 nm P450 BM3 M11 or CYP1A2 in 100 mm potassium phosphate buffer (pH 7.4) supplemented with 5 mm MgCl_2_ and 2 mm EDTA. Seven substrate concentrations ranging from 10 to 750 μm were used in a final incubation volume of 100 μL. Reaction mixtures were preincubated in a shaking water bath set at the incubation temperature for 10 min. Reactions were initiated by addition of 10 % (*v*/*v*) of a prewarmed solution containing NADPH regenerating system; final concentrations were 0.5 mm NADPH, 10 mm glucose 6‐phosphate, and 0.4 units mL^−1^ glucose‐6‐phosphate dehydrogenase. The reaction was allowed to proceed for 4 min at different temperatures and then stopped by the addition of 100 μL ice‐cold methanol. The denatured enzyme fractions were precipitated by centrifugation for 20 min at 14 000 rpm (20 817 *g*). The supernatants were isolated and analyzed by HPLC or LC‐MS as described below.

For mefenamic acid and M11 enzyme kinetic parameters for the formation of the three metabolites were determined at eight temperatures ranging from 4 to 45 °C, to investigate the linearity in more detail. The other substrates were incubated at four temperatures ranging from 4 to 35 °C. The enzyme kinetic parameters *V*_max_ and *K*_M_ were determined by nonlinear regression according to the Michaelis–Menten equation using GraphPad Prism 7.0 software (GraphPad, San Diego, CA, USA).

Δ and ln (*A*_1_ */A*_2_) in Equation (5) for competing enzyme reactions were determined by plotting the logarithms of the ratio of the *V*_max_ values measured at different temperatures against the inverse of the absolute temperature *T*. According to Equation (5), the slope of this curve, when linear, corresponds to the sum Δ of ΔΔ*G*_bind_ and Δ*E*_a_ divided by the negative gas constant *R* (8.3145 J mol^−1^ K^−1^). The slopes and intercepts of the modified Arrhenius plots were determined by linear regression using the GraphPad Prism 7.0 software.

**Determination of competitive intermolecular kinetic isotope effects of hydroxylation of mefenamic acid**: A method for full deuteration of mefenamic acid was described previously^68^ and used to synthesize deuterated mefenamic acid and to study kinetic isotope effects for its conversion by BM3 M11 (Figure 3). Repeated cycles of microwave‐assisted H/D exchange in presence of platinum and palladium catalysts resulted in a 52:48 % mixture of [D_13_]mefenamic acid and [D_12_]mefenamic acid. Because all three metabolites showed a 52:48 % ratio of D_12_ and D_11_ degrees of labelling, it was concluded that the remaining hydrogen‐atom was localized at a position not corresponding to the SOMs. Therefore, the metabolic incubations were performed using the mixture of [D_13_]mefenamic acid and [D_12_]mefenamic acid.

Because the deuterated compound contained low levels of unidentified side products, the kinetic isotope effects were determined in a competitive intermolecular experiment with equimolar mixtures of mefenamic acid and [D_12_,_13_]mefenamic acid, so that eventual effects of these side products on the reactions were applicable to both labelled and unlabeled mefenamic acid to the same extent.^30^ All incubations were performed with 100 nm P450 in 100 mm potassium phosphate buffer (pH 7.4) supplemented with 5 mm MgCl_2_ and 2 mm EDTA. Total substrate concentrations of the mixture of labelled and unlabeled mefenamic acid were 75 and 750 μm. Reaction mixtures (total volume 100 μL) were pre‐warmed at the incubation temperature for 10 min, before initiating the reaction by addition of a NADPH regenerating system (final concentrations of 0.5 mm NADPH, 10 mm glucose 6‐phosphate, and 0.4 unit mL^−1^ glucose‐6‐phosphate dehydrogenase). The reaction was allowed to proceed for 4 min at different temperatures and then stopped by the addition of 100 μL ice‐cold methanol. The protein was removed by centrifugation for 20 min at 14 000 rpm (20 817 *g*). The supernatants were analyzed on an Agilent 1200 series rapid resolution LC equipped with a TOF Agilent 6230 mass spectrometer (Agilent technologies, Waldbronn, Germany). Data processing was performed with the Mass Hunter Qualitative Analysis software package (version B.06.00). Note that the extracted ion chromatograms of the deuterated hydroxy metabolites and substrate showed slightly shorter retention times than their non‐deuterated counterparts, indicating that the lipophilicity was slightly reduced upon deuteration. Assuming that the deuteration of the aromatic rings and methylene group does not affect the ionization efficiency of the metabolites, the kinetic isotope effect of full deuteration were for all three metabolites directly calculated from the peak areas in the ion chromatograms. Because kinetic isotope effects were studied at a single concentration, no Arrhenius plots were constructed because activities did not represent *V*_max_ values.

**Analytical methods**: The analyses of metabolites were performed by reversed‐phase liquid chromatography using a Shimadzu HPLC equipped with two LC‐ 20AD pumps, a SIL20AC autosampler and a SPD20A UV detector. Lab Solution software of Shimadzu was used to control the HPLC‐system, data acquisition and data analysis. For metabolite identification and quantification of isotope ratios in the competitive isotope experiment, an Agilent 1200 series rapid resolution LC was used which was connected to an Agilent 6230 time‐of‐flight mass spectrometer equipped with an electrospray ionization (ESI) source operating in positive ion mode. A capillary voltage of 3500 V was used, and nitrogen was used both as drying gas (10 L min^−1^) and nebulizing gas (pressure 50 psig) at a constant gas temperature of 350 °C; 1000 MS spectra per second were acquired and analysis was performed using Agilent MassHunter Qualitative analysis software (version 2.0).

For all compounds, a Luna 5 μm C_18_ column (4.6×150 mm) was used as stationary phase and gradients were constructed by using two mobile phases: eluent A (0.8 % acetonitrile, 99 % water, 0.2 % formic acid) and eluent B (0.8 % water, 99 % acetonitrile, 0.2 % formic acid).

For the analysis of metabolites of mefenamic acid, the first 5 min was isocratic at 40 % eluent B. From 5 until 30 min, the concentration of eluent B was increased linearly to 100 %, followed by linear decrease back to 40 % between 30 and 30.5 min. Isocratic re‐equilibration at 40 % eluent B was maintained until 45 min. The flow rate was 0.5 mL min^−1^. UV/Vis detection was performed at 254 nm.

For the analysis of metabolites of testosterone, the first 1 min was isocratic at 50 % eluent B. From 1 to 20 min the percentage of eluent B was increased linearly to 99 %; and from 20 to 20.5 min linearly decreased to 50 % B and maintained at 50 % for re‐equilibration until 30 min. The flow rate was 0.5 mL min^−1^. UV/Vis detection was performed at 254 nm.

**Molecular dynamics (MD) simulations of substrate binding to P450 BM3 M11 and CYP1A2**: MD simulations were carried out to quantify the occurrences of different catalytically active binding poses over time, as an (entropic) measure of the frequency or efficiency of collisions allowing for transition state formation. Simulations were carried out both for mefenamic acid and testosterone, either bound to BM3 M11 or CYP1A2. To define occurrence of binding poses that are suitable for hydroxylation of either an aliphatic or aromatic C−H moiety, we used geometric criteria for transition state formation as reported by Mulholland and co‐workers.^63^ These criteria are similar as we used before and were as before extended with a rule to exclude assignment of conformations to be catalytically active for aromatic hydroxylation, in case the angle between the C−H site‐of‐metabolism bond and the vector connecting the corresponding hydrogen with the ferryl oxygen was between 140° and 220° (Table 7), in order to account for the possible detrimental effect of hydrogen interposition on C−H activation by the ferryl oxygen.^32^ Thus, a given enzyme–substrate conformation was identified as a catalytically active pose and suitable for transition state formation when fulfilling the criteria summarized in Table 7.

**Table 7:** Geometric criteria to identify conformations from molecular dynamics simulations as catalytically active binding poses for hydroxylation of aromatic or aliphatic C−H sites of metabolism (SOMs). Unless noted otherwise, these criteria were derived from combined QM/MM studies of Mulholland and co‐workers.^63^

Type of hydroxylation	Distance criteria	Angle criteria
aromatic	distance carbon of site of metabolism to ferryl oxygen	C_SOM_‐H_SOM_‐O_Fe_ angle should not be between 140° and 220°^[a]^
	(C_SOM_‐O_Fe_)<0.35 nm	
aliphatic	distance of hydrogen of site of metabolism to ferryl oxygen	H_SOM_‐O_Fe_‐Fe angle should be between 110° and 130°
	(H_SOM_‐O_Fe_)<0.35 nm

[a] Adapted from ref. 32.

For BM3 M11, chain B of the crystal structure of the heme domain of mutant BM3 M11 (PDB ID: 5E9Z)^32^ was used as template for docking and subsequent MD simulations. Missing residue Q73 and missing atoms of residues K31, Q73, K94, K97, Q109, Q110, D136, K187, K218, Q229, T245, R255, Q288, K306, K449 were added with Modeller 9.3.^69^ For docking and MD simulations with CYP1A2, the crystal structure from PDB ID: 2HI4 was used.^70^ To obtain protein‐binding poses for MF and TE to start MD simulations from, they were docked into the protein templates (using the PLANTS docking software, version 1.2^71^ and the ChemPLP scoring function^72^) and equilibrated in MD simulations in which the heme group was described in its resting state (i.e., with a ferryl‐oxygen dummy atom). Prior to docking, initial (steepest‐descent) energy minimization of MF and TE with the MMFF94 force field was performed using MOE.^73^ After docking and MD with the heme modeled in the resting state, enzyme‐substrate conformations consistent with 3′‐methyl hydroxylation (mefenamic acid in BM3 M11), 4′‐ and 5‐hydroxylation (mefenamic acid in BM3 M11 and CYP1A2), and 16β‐hydroxylation (testosterone in BM3 M11) were selected to start MD simulations from, in which the heme group was modelled in its compound I state. For this purpose, two starting poses for MD were selected per combination of mutant and product formation using the geometric criteria in Table 7.^32^ For MD simulations of testosterone in its catalytically active pose for 15β/16β‐OH‐testosterone formation, a single starting pose was selected from which three independent MD simulations were started. All MD simulations (including thermal equilibration and 100 ns production simulations) were performed using identical simulation settings and force‐field parameters for the protein and heme group (either in the resting or the compound I state) as described in ref. 74. Atomic coordinates were written out to disk every 100 ps. Partial atomic charges of MF and TE for use in MD were obtained with GAMESS (Version 1 May 2012)^75^ at the Hartree–Fock level using the 6‐31G* basis set. Other interaction parameters for MF and TE were used from the General Amber Force Field for organic molecules version 1.7.^76^ After MD, protein‐ligand interaction profiles during simulation were analyzed in terms of protein residue‐ligand interaction frequencies using the dedicated Python‐based biomolecular analysis library MDInteract, which is freely available at https://github.com/MD‐Studio/MDInteract.^77^

## Conflict of interest

*The authors declare no conflict of interest*.

## Supporting information

As a service to our authors and readers, this journal provides supporting information supplied by the authors. Such materials are peer reviewed and may be re‐organized for online delivery, but are not copy‐edited or typeset. Technical support issues arising from supporting information (other than missing files) should be addressed to the authors.

Supplementary
