# Supplementary material for: A Modified Arrhenius Approach to Thermodynamically Study Regioselectivity in Cytochrome P450‐Catalyzed Substrate Conversion
Source: Chembiochem. 2020 Feb 25;21(10):1461–72. doi: 10.1002/cbic.201900751 (PMC7318578; doi:10.1002/cbic.201900751)
Supplement: Supplementary file 1 — Supplementary [file CBIC-21-1461-s001.pdf]

CHEMBIOCHEM

Supporting Information

A Modified Arrhenius Approach to Thermodynamically Study Regioselectivity in Cytochrome P450-Catalyzed Substrate Conversion

Rosa A. Luirink+, Marlies C. A. Verkade-Vreeker+, Jan N. M. Commandeur, and Daan P. Geerke*[a]

cbic_201900751_sm_miscellaneous_information.pdf

Table S1:

Temperature dependence of enzyme kinetic parameters for the regioselective hydroxylation of testosterone by P450 BM3 M11 (at either the 2β, 15β or 16β position, Figure 3).

	2β-hydroxy testosterone			15β-hydroxy testosterone			16β-hydroxy testosterone		
T (K)	K_m (μM)	V_{max}[a]	V_{max}/K_m[b]	K_m (μM)	V_{max}[a]	V_{max}/K_m[b]	K_m (μM)	V_{max}[a]	V_{max}/K_m[b]
279.4	151 ± 34	1.4 ± 0.1	9.3	96 ± 23	7.1 ± 0,6	74	94 ± 20	3.9 ± 0.3	42
290.6	47 ± 14	3.9 ± 0.3	83	38 ± 10	20 ± 1.5	529	35 ± 8	7.9 ± 0.5	226
299.7	42 ± 10	4.7 ± 0.5	112	37 ± 8	20 ± 1.2	530	54 ± 19	6.6 ± 0.4	122
309.6	59 ± 14	6.6 ± 0.7	112	46 ± 9	26 ± 2,0	554	31 ± 6	7.0 ± 0.6	226

a. Unit: nmol product/min /nmol enzyme;
b. Unit: μL/min/nmol enzyme.

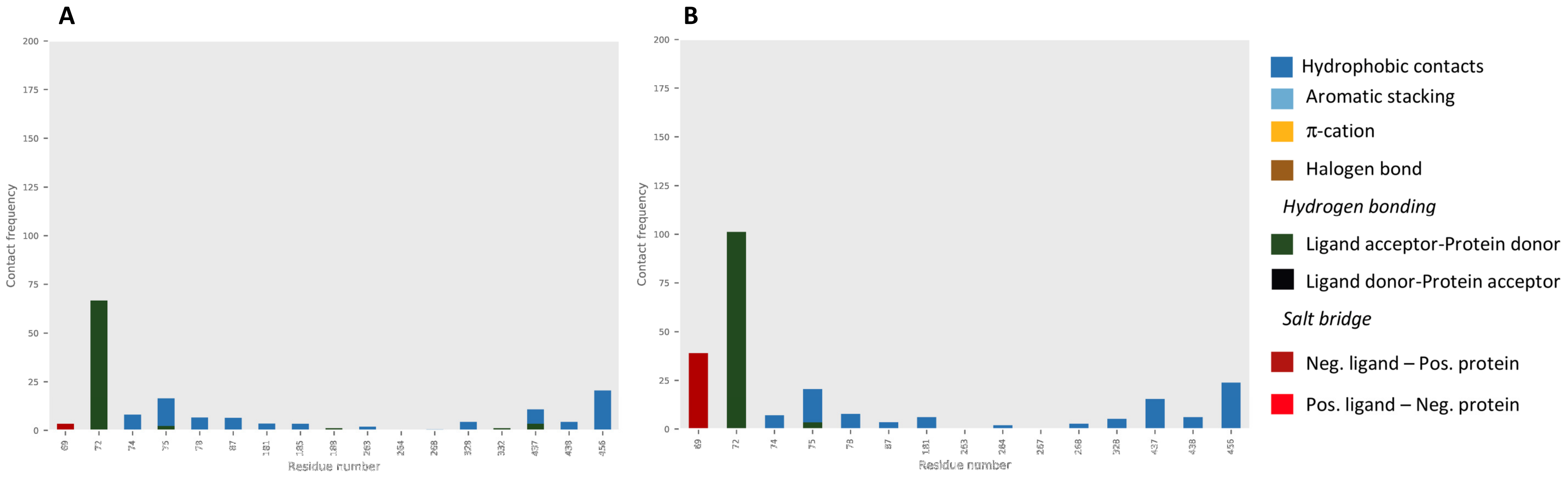

Figure S1.

Interaction profiles of mefenamic acid during the two independent MD simulations (A and B) of P450 BM3 M11 in complex with mefenamic acid bound in an orientation compatible with 3’ methyl hydroxylation. Percental contact frequencies for each protein residue-ligand interaction are represented by vertically stacked bars where the bar colors correspond to a specific interaction type as listed in the graph legend. Hydrophobic contact frequencies are divided by 10 because of their relative abundance with respect to the other classified interactions.

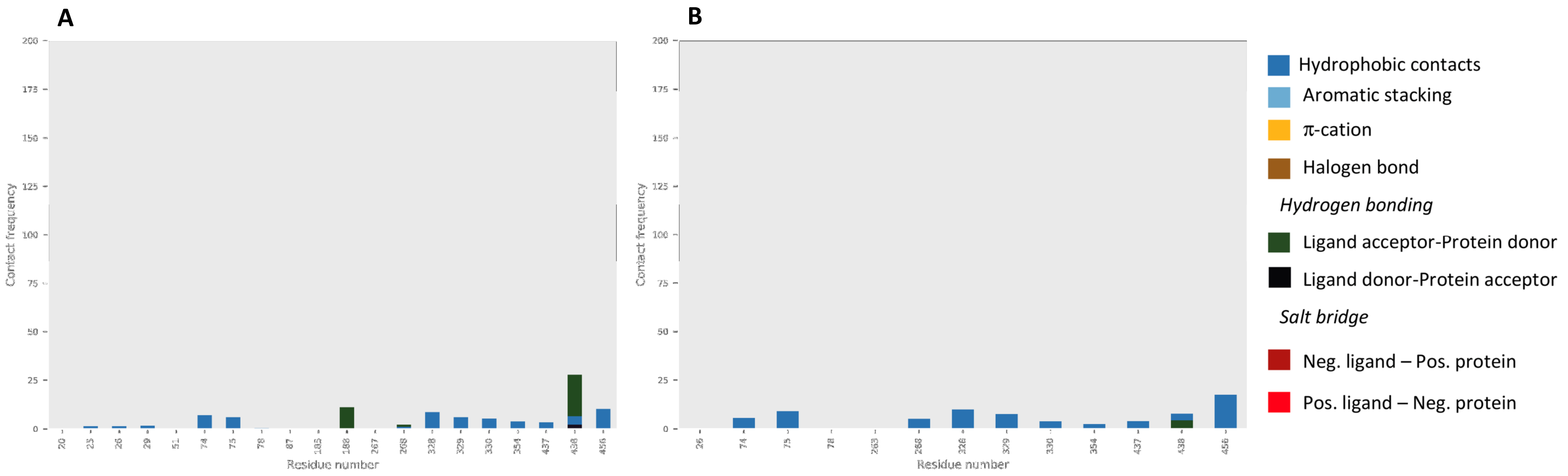

Figure S2.
Interaction profiles of mefenamic acid during the two independent MD simulations (A and B) of P450 BM3 M11 in complex with mefenamic acid bound in an orientation compatible with C5 hydroxylation. Percental contact frequencies for each protein residue-ligand interaction are represented by vertically stacked bars where the bar colors correspond to a specific interaction type as listed in the graph legend. Hydrophobic contact frequencies are divided by 10 because of their relative abundance with respect to the other classified interactions.

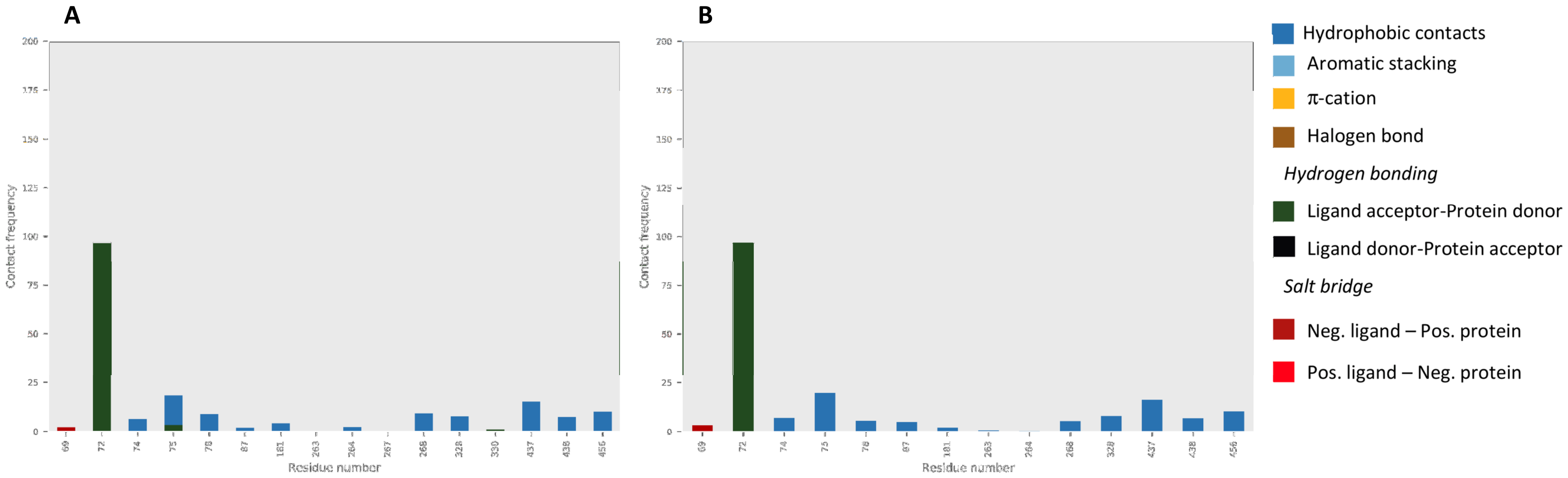

Figure S3.
Interaction profiles of mefenamic acid during the two independent MD simulations (A and B) of P450 BM3 M11 in complex with mefenamic acid bound in an orientation compatible with C4' hydroxylation. Percental contact frequencies for each protein residue-ligand interaction are represented by vertically stacked bars where the bar colors correspond to a specific interaction type as listed in the graph legend. Hydrophobic contact frequencies are divided by 10 because of their relative abundance with respect to the other classified interactions.

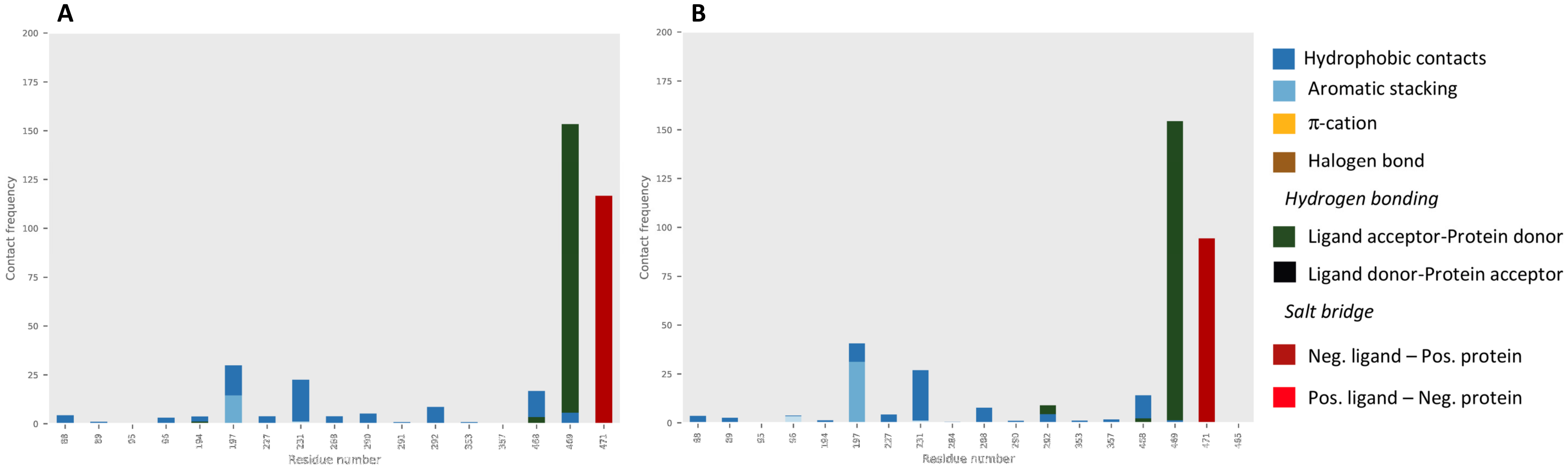

Figure S4.
Interaction profiles of mefenamic acid during the two independent MD simulations (A and B) of CYP1A2 in complex with mefenamic acid bound in an orientation compatible with C5 hydroxylation. Percental contact frequencies for each protein residue-ligand interaction are represented by vertically stacked bars where the bar colors correspond to a specific interaction type as listed in the graph legend. Hydrophobic contact frequencies are divided by 10 because of their relative abundance with respect to the other classified interactions.

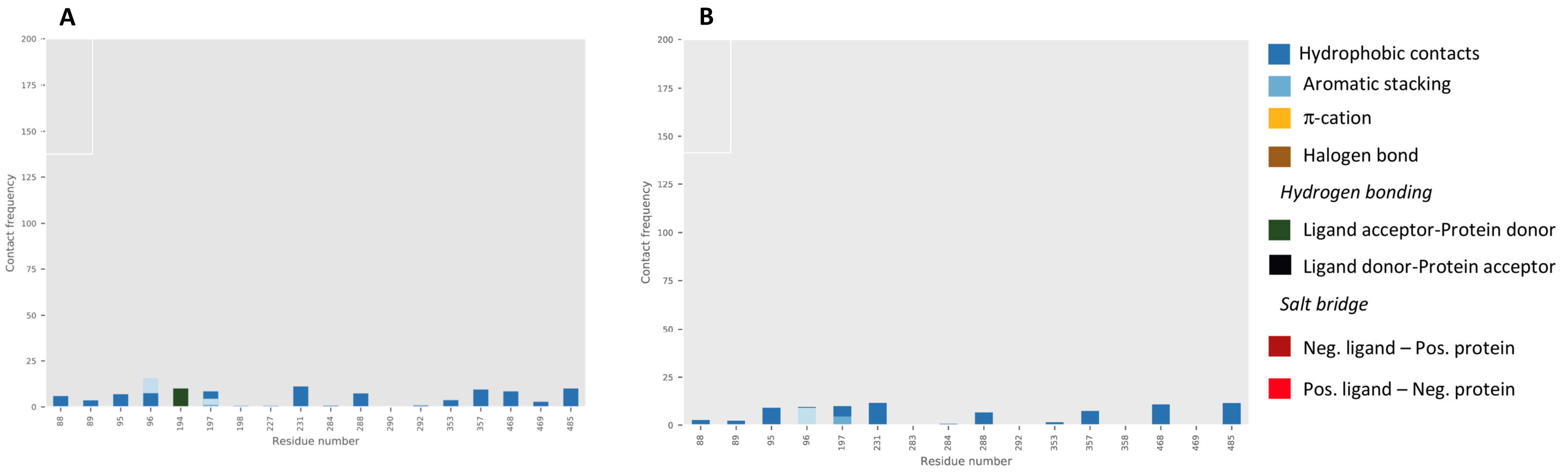

Figure S5.
Interaction profiles of mefenamic acid during the two independent MD simulations (A and B) of CYP1A2 in complex with mefenamic acid bound in an orientation compatible with C4’ hydroxylation. Percental contact frequencies for each protein residue-ligand interaction are represented by vertically stacked bars where the bar colors correspond to a specific interaction type as listed in the graph legend. Hydrophobic contact frequencies are divided by 10 because of their relative abundance with respect to the other classified interactions.
